# Supplementary material for: Tracking changes in SARS-CoV-2 transmission with a novel outpatient sentinel surveillance system in Chicago, USA
Source: Nat Commun. 2022 Sep 22;13:5547. doi: 10.1038/s41467-022-33317-6 (PMC9499975; doi:10.1038/s41467-022-33317-6)
Supplement: Supplementary file 1 — Supplementary Information [file 41467_2022_33317_MOESM1_ESM.pdf]

Supplement for:

Tracking changes in SARS-CoV-2 transmission with a novel outpatient sentinel surveillance system in Chicago, USA

Reese Richardson[1,2], Emile Jorgensen[2], Philip Arevalo[3], Tobias M. Holden[4], Katelyn M. Gostic[3], Massimo Pacilli[2], Isaac Ghinai[2], Shannon Lightner[5], Sarah Cobey[3], Jaline Gerardin[4]*

[1] Department of Chemical and Biological Engineering, Northwestern University, Evanston IL

[2] Chicago Department of Public Health, Chicago IL

[3] Department of Ecology and Evolution, University of Chicago, Chicago IL

[4] Department of Preventive Medicine and Institute for Global Health, Northwestern University, Chicago IL

[5] Illinois Department of Public Health, Springfield IL

*To whom correspondence should be addressed: jgerardin@northwestern.edu

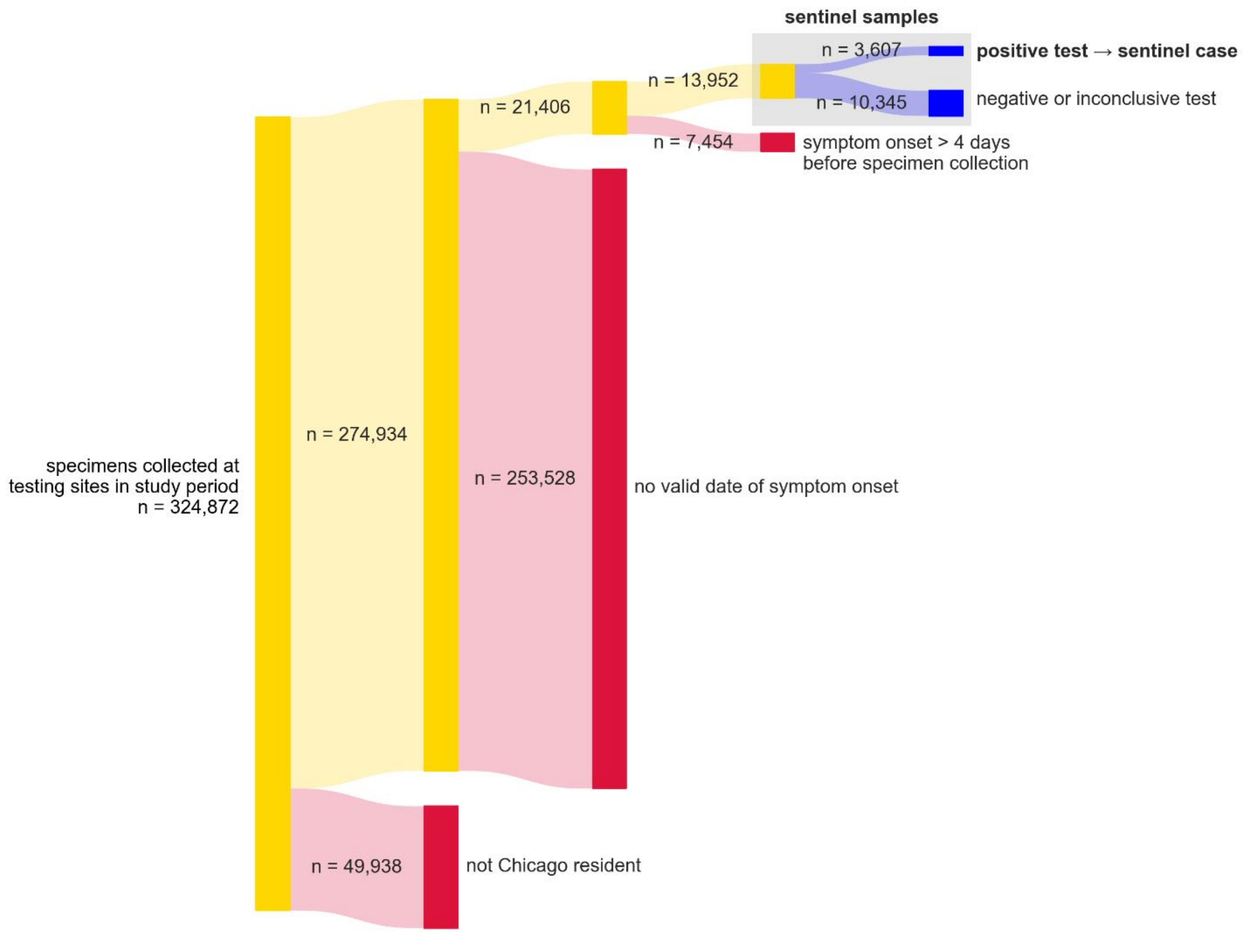

Figure S1: Sankey diagram depicting selection of sentinel samples and sentinel cases over the study period (September 27, 2020 to June 13, 2021).

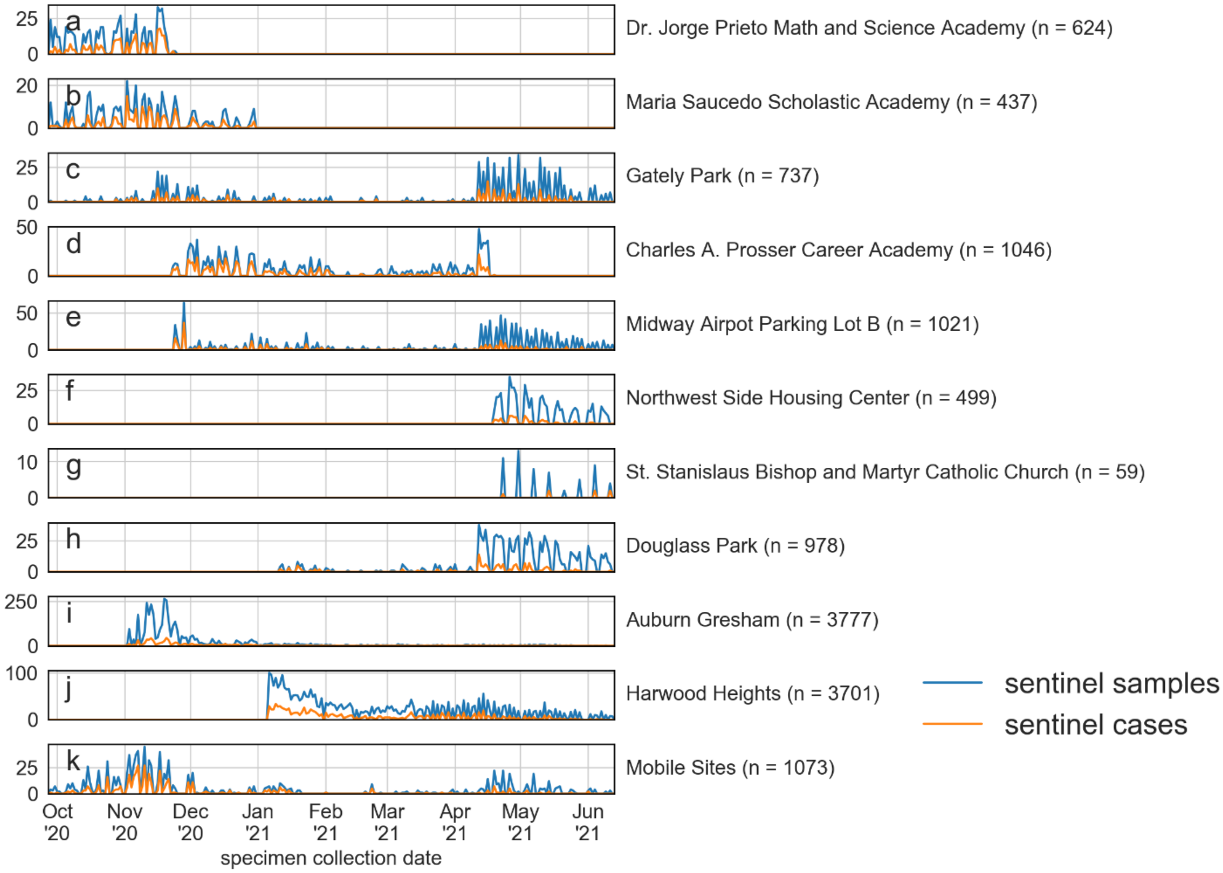

Figure S2: Daily sentinel sample and sentinel case counts by sentinel test site.

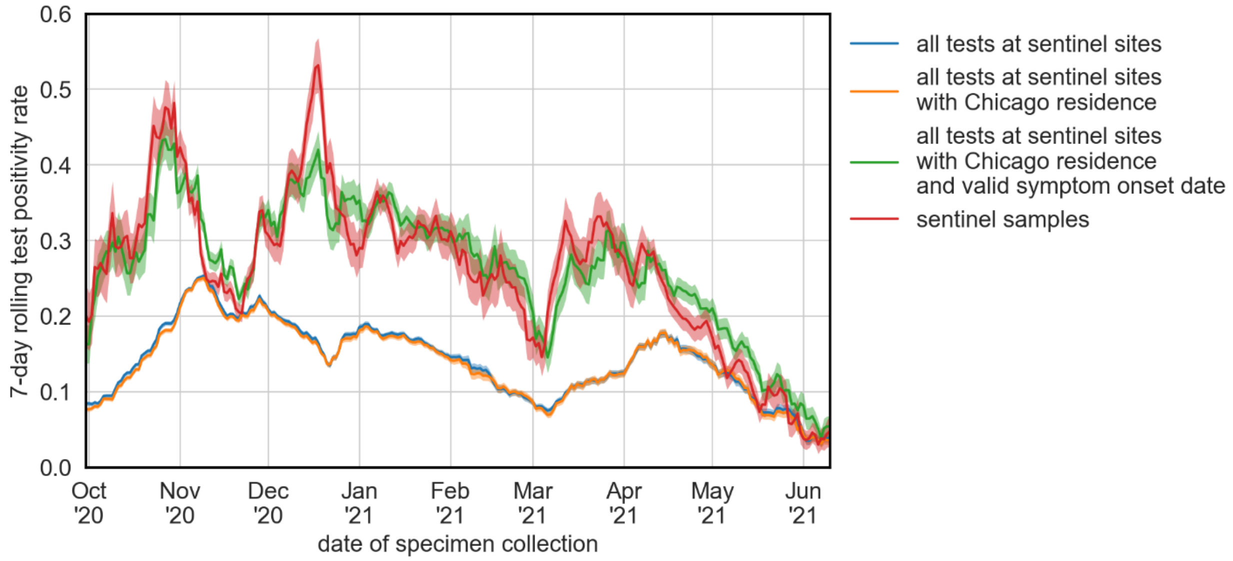

Figure S3: 7-day rolling positivity rates among all tests collected at sentinel sites (blue), all tests collected at sentinel sites with Chicago residence (orange), all tests collected at sentinel sites with Chicago residence and valid symptom onset date (green), and sentinel samples (red) during study period (September 27, 2020 to Jun 13, 2021). Solid lines indicate nominal proportions and shaded regions show ±1 standard deviation of the sample proportion. See **Figure S1** for Sankey diagram describing selection of sentinel samples.

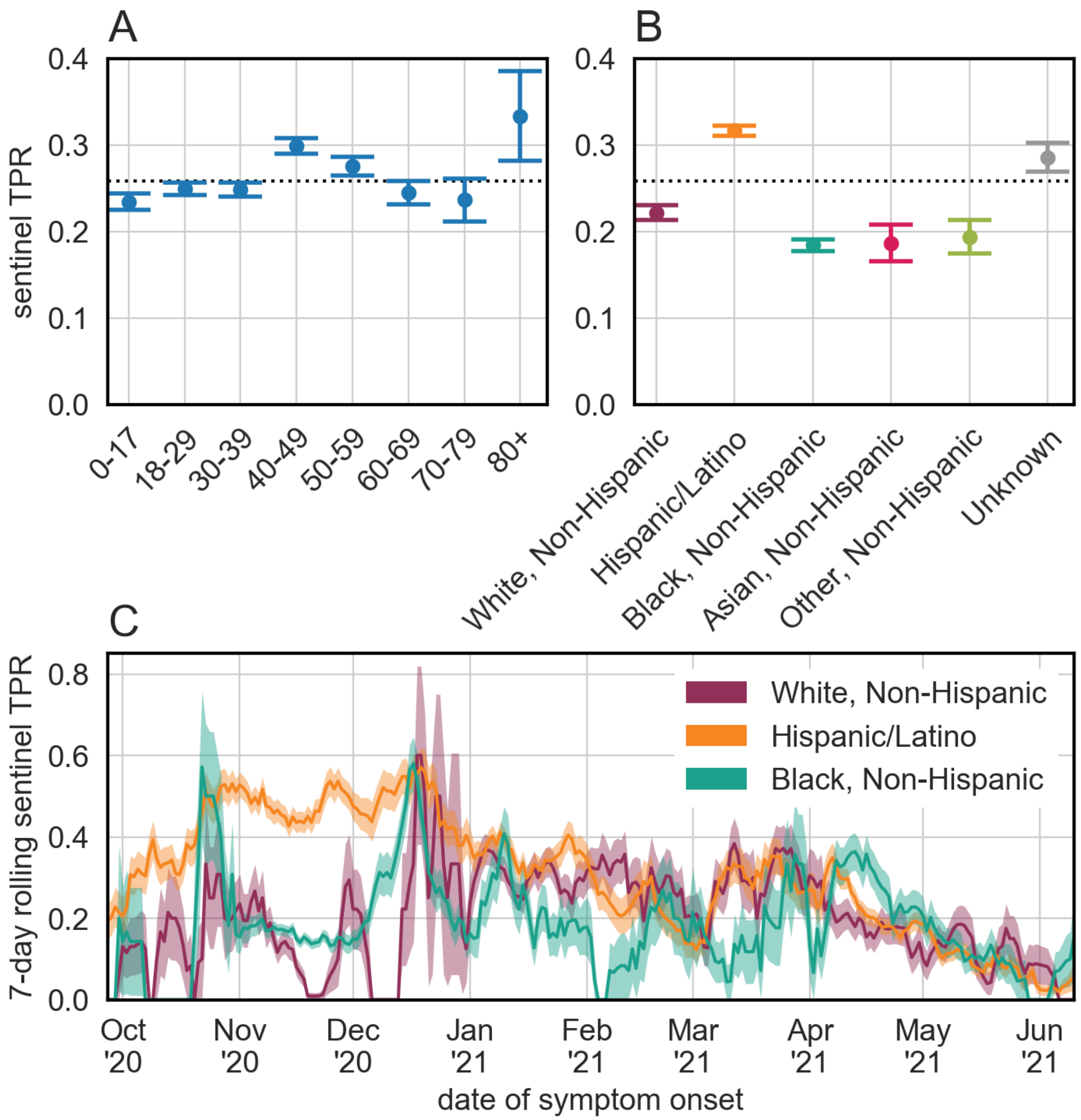

Figure S4: Sentinel test positivity rates (TPR) by age and race/ethnicity over the study period. **(A)** Sentinel TPR by age group over the study period. Dots indicate nominal proportions. Error bars indicate ±1 standard deviation of the proportion. Horizontal dashed line represents sentinel TPR across all age groups (25.8%). **(B)** Sentinel TPR by race/ethnicity over the study period. Error bars indicate the sample proportion ±1 standard deviation of the proportion. Horizontal dashed line represents sentinel TPR across all racial/ethnic groups (25.8%). **(C)** Weekly rolling sentinel TPR by race/ethnicity over the study period. Solid lines indicate nominal proportions. Shaded regions indicate the sample proportion ±1 standard deviation of the proportion. Non-Hispanic Asian, Non-Hispanic Other, and Unknown are excluded from this plot due to low weekly counts.

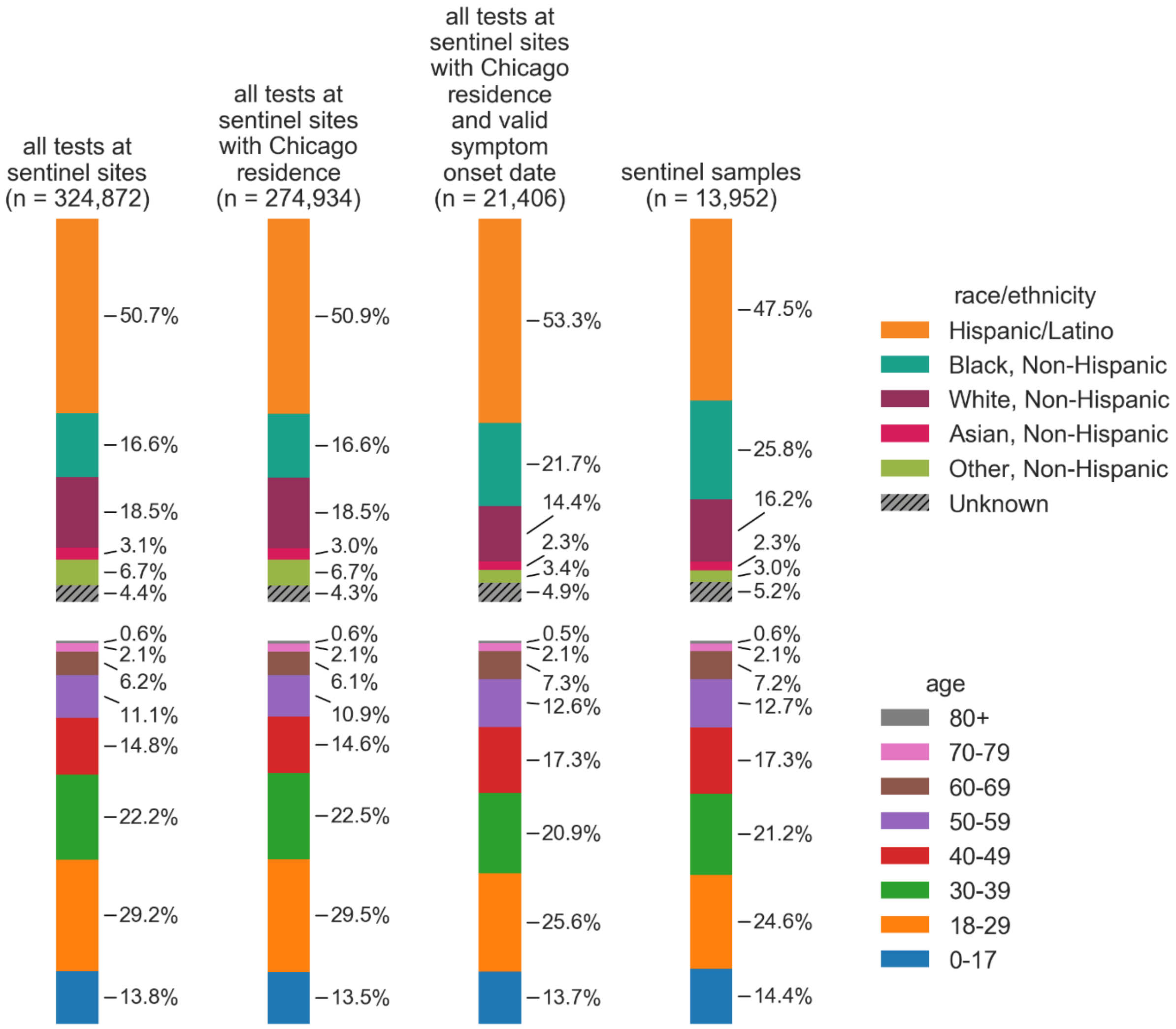

Figure S5: Demographic breakdowns by race/ethnicity and age group among all tests collected at sentinel sites, all tests collected at sentinel sites with Chicago residence, all tests collected at sentinel sites with Chicago residence and valid symptom onset date, and sentinel samples during study period (September 27, 2020 to Jun 13, 2021). See **Figure S1** for Sankey diagram describing selection of sentinel samples.

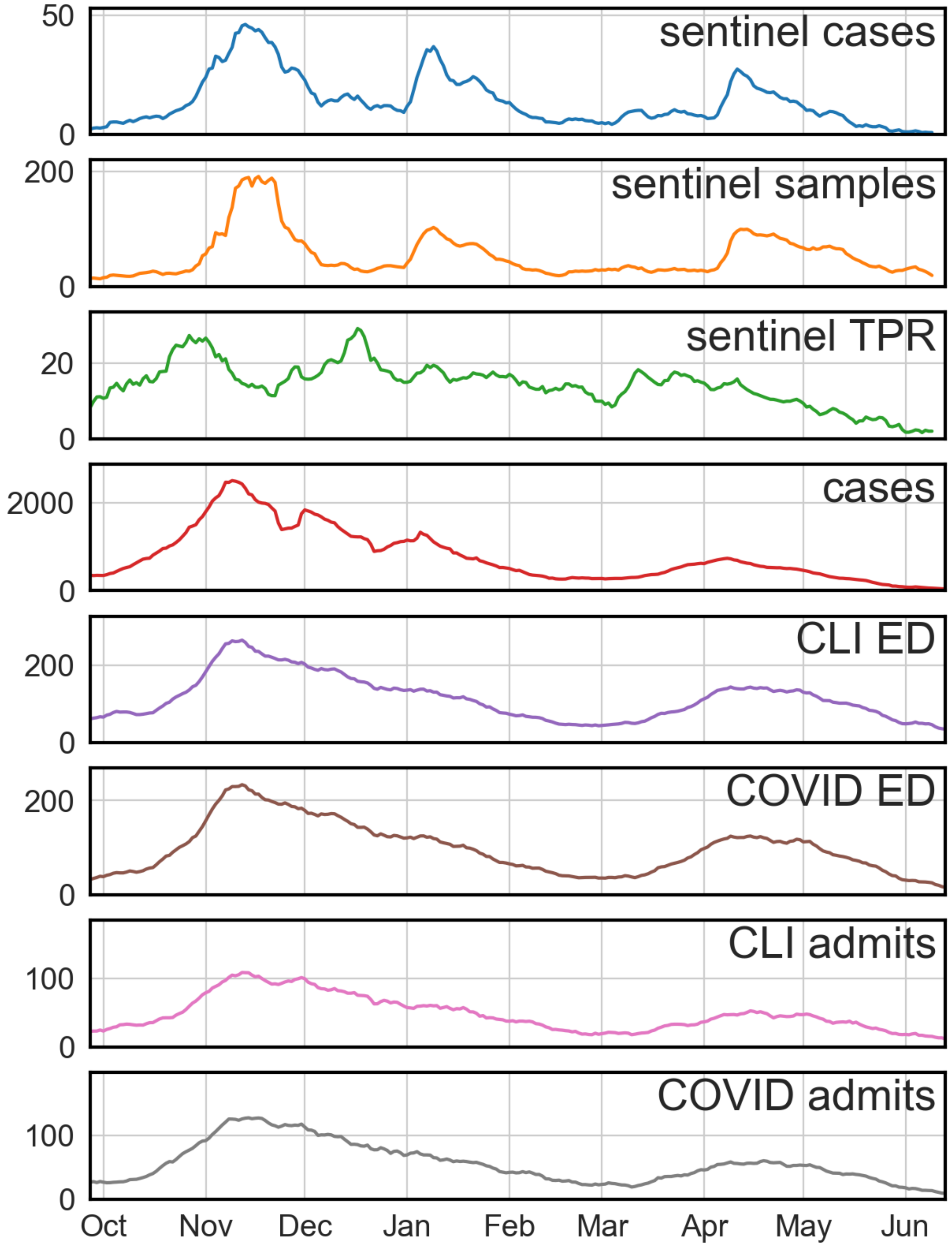

Figure S6: 7-day rolling averages of indicators used. See **Methods** for definitions of indicators.

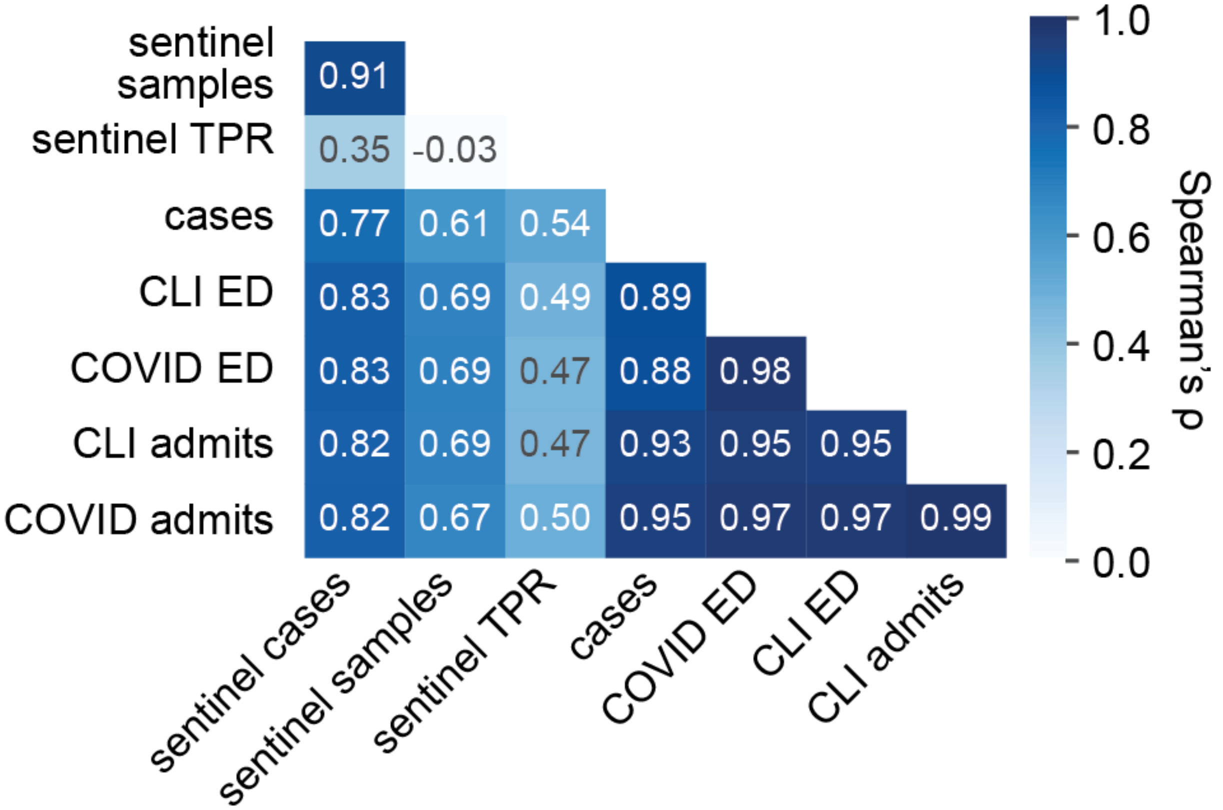

Figure S7: Spearman's ρ correlation matrix between $R(t)$ series. See **Fig 4B.**

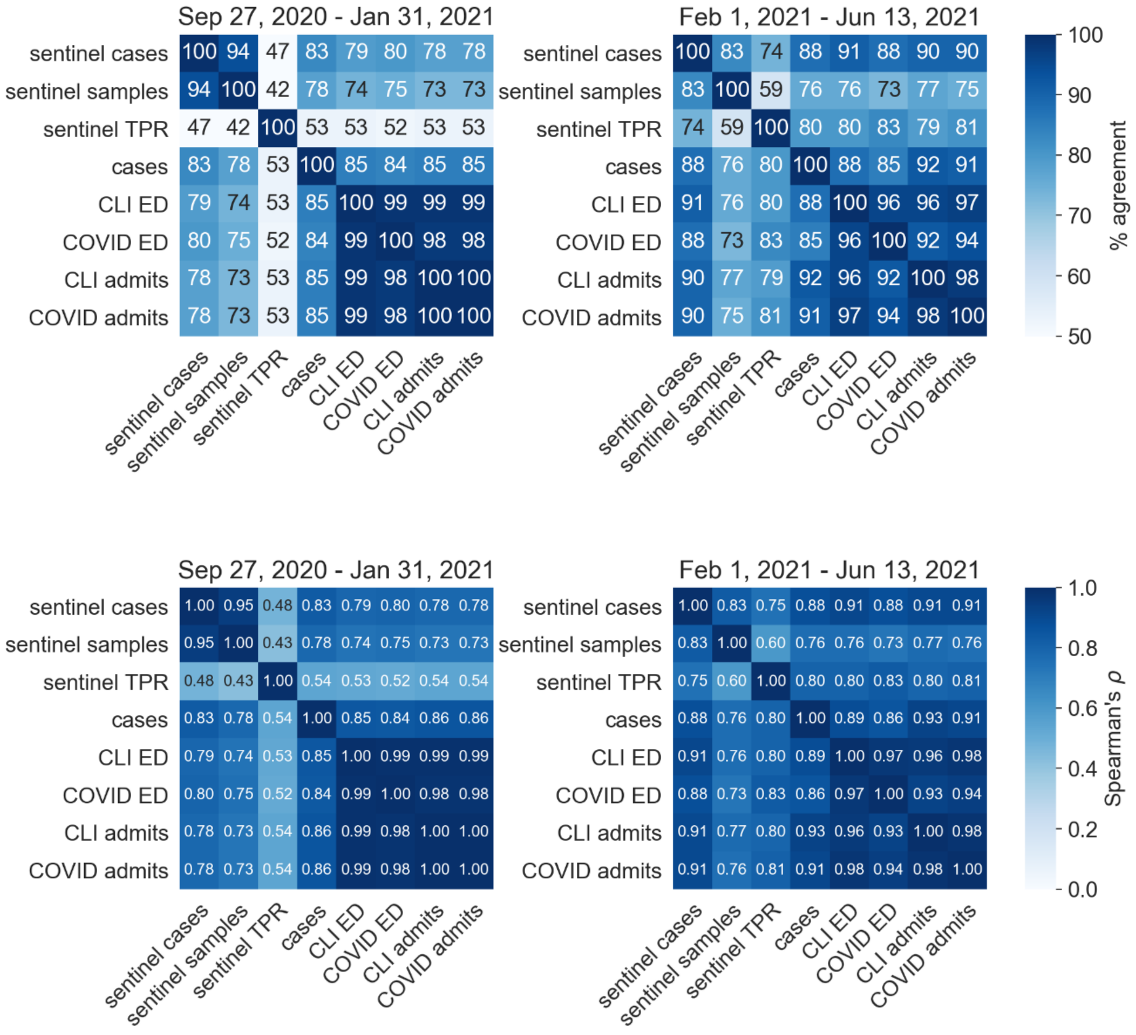

Figure S8: Similarity matrices of percent agreement and Spearman's ρ between each pairwise comparison of $R(t)$ series for the front half (Sep 27, 2020 – Jan 31, 2021) and back half (Feb 1, 2021 – Jun 13, 2021) of the study window. Percent agreement is the percentage of dates when the median $R(t)$ estimates of two series are both ≥ 1.0 or both < 1.0. Compare to **Figure 4B.**

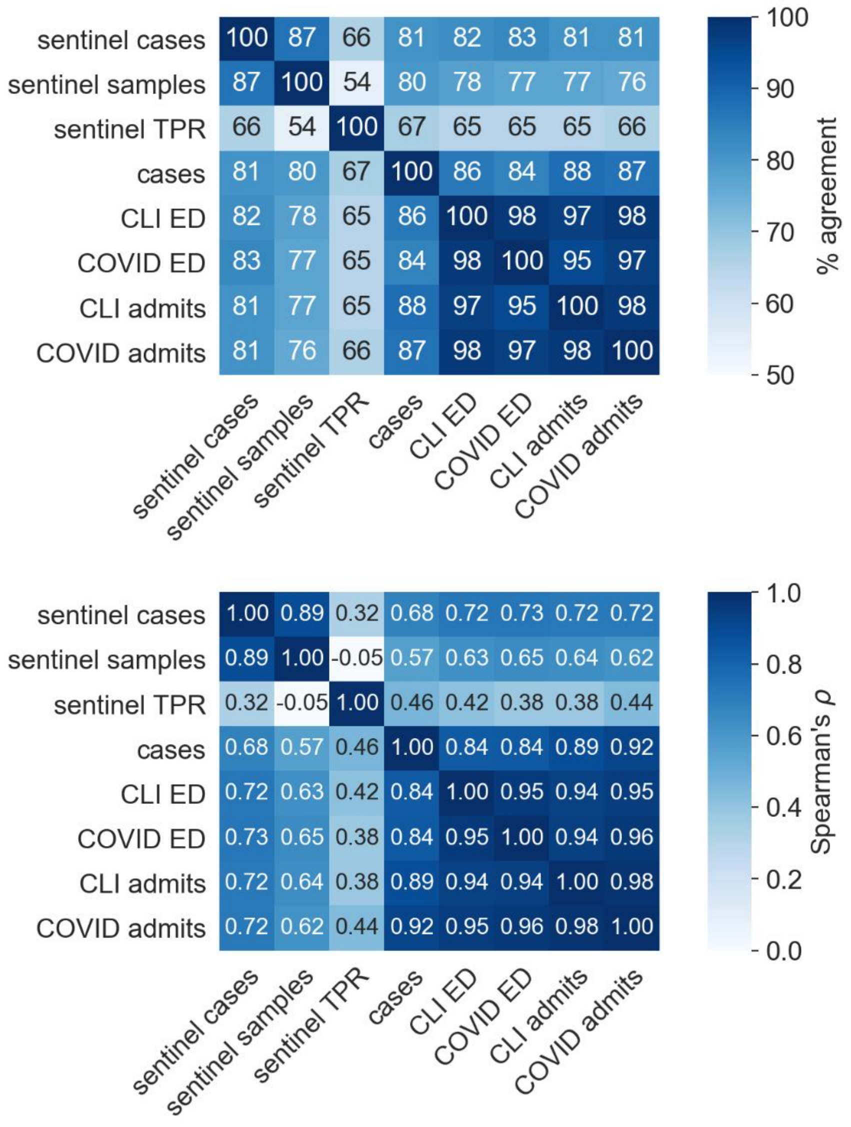

Figure S9: Similarity matrix of percent agreement and Spearman's ρ between each pairwise comparison of *R(t)* series, calculated with a seven-day smoothing window (as opposed to a 14-day smoothing window, as presented in **Figure 4B)**. Percent agreement is the percentage of dates when the median $R(t)$ estimates of two series are both ≥ 1.0 or both < 1.0.

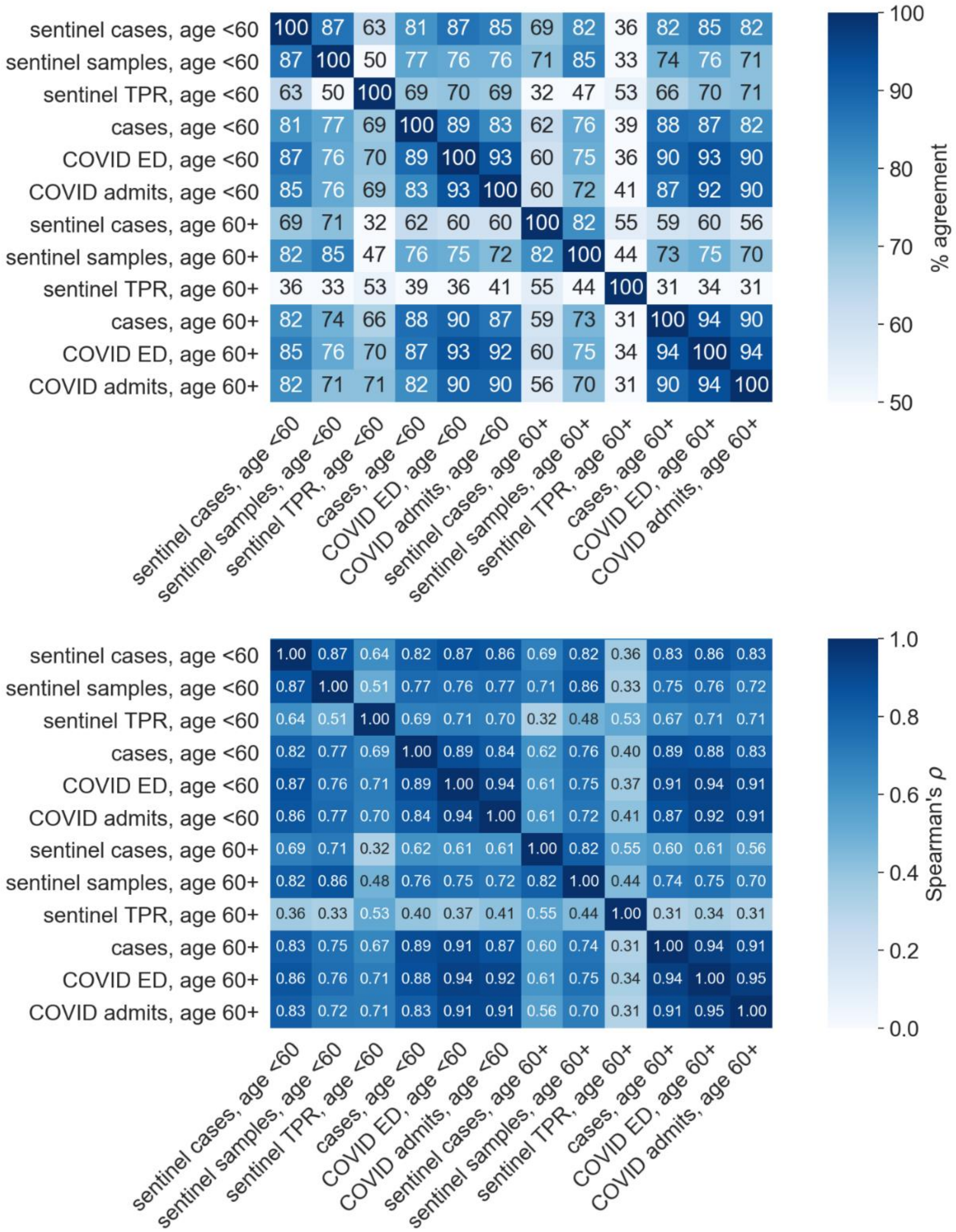

Figure S10: Similarity matrix of percent agreement and Spearman's ρ between $R(t)$ series generated from indicators stratified at age 60. Percent agreement is the percentage of dates when the median $R(t)$ estimates of two series are both ≥ 1.0 or both < 1.0. The lower agreement between sentinel cases $R(t)$ age 60+ and other indicators ages 60+ is likely the product of the low volume of sentinel samples in this age group (only 9.9% of sentinel samples were age 60+, see **Figure 3B**).

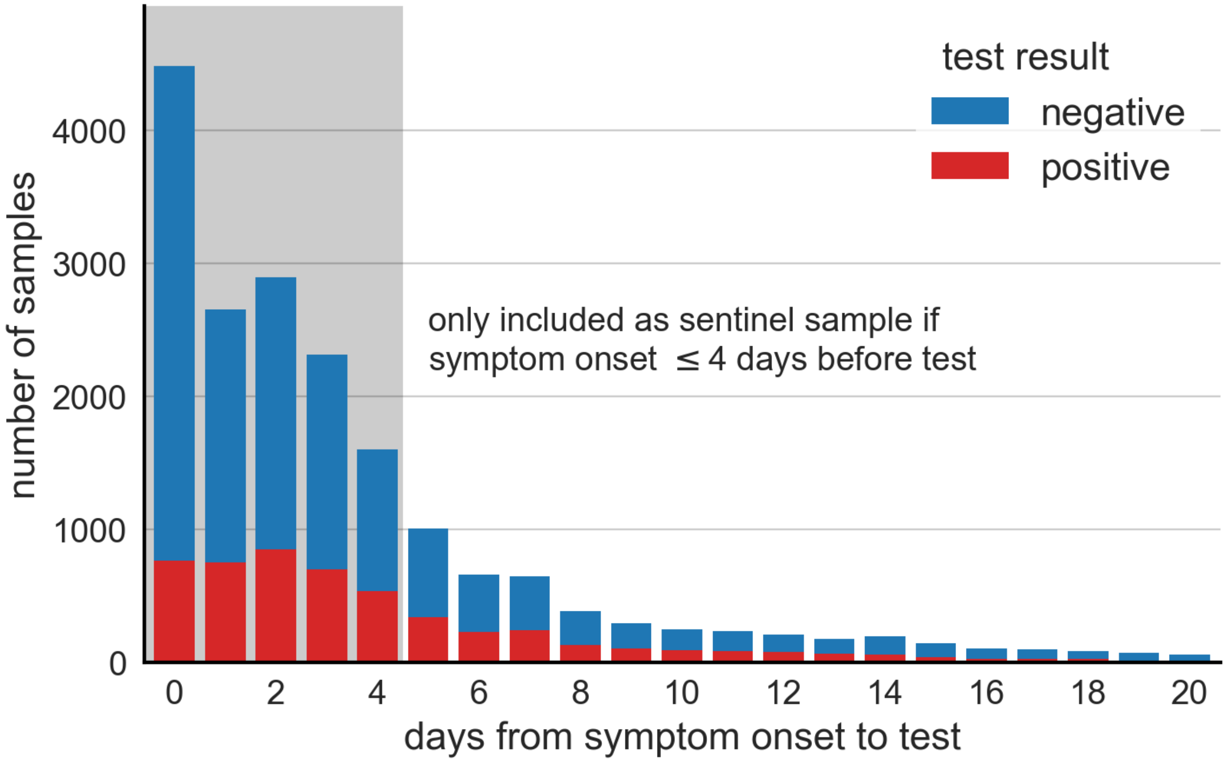

Figure S11: Distribution of specimens eligible to be sentinel samples by days elapsed between reported date of symptom onset and date of specimen collection. Time between reported date of symptom onset and specimen collection was greater than 20 days for 2,804 specimens (13.1%).

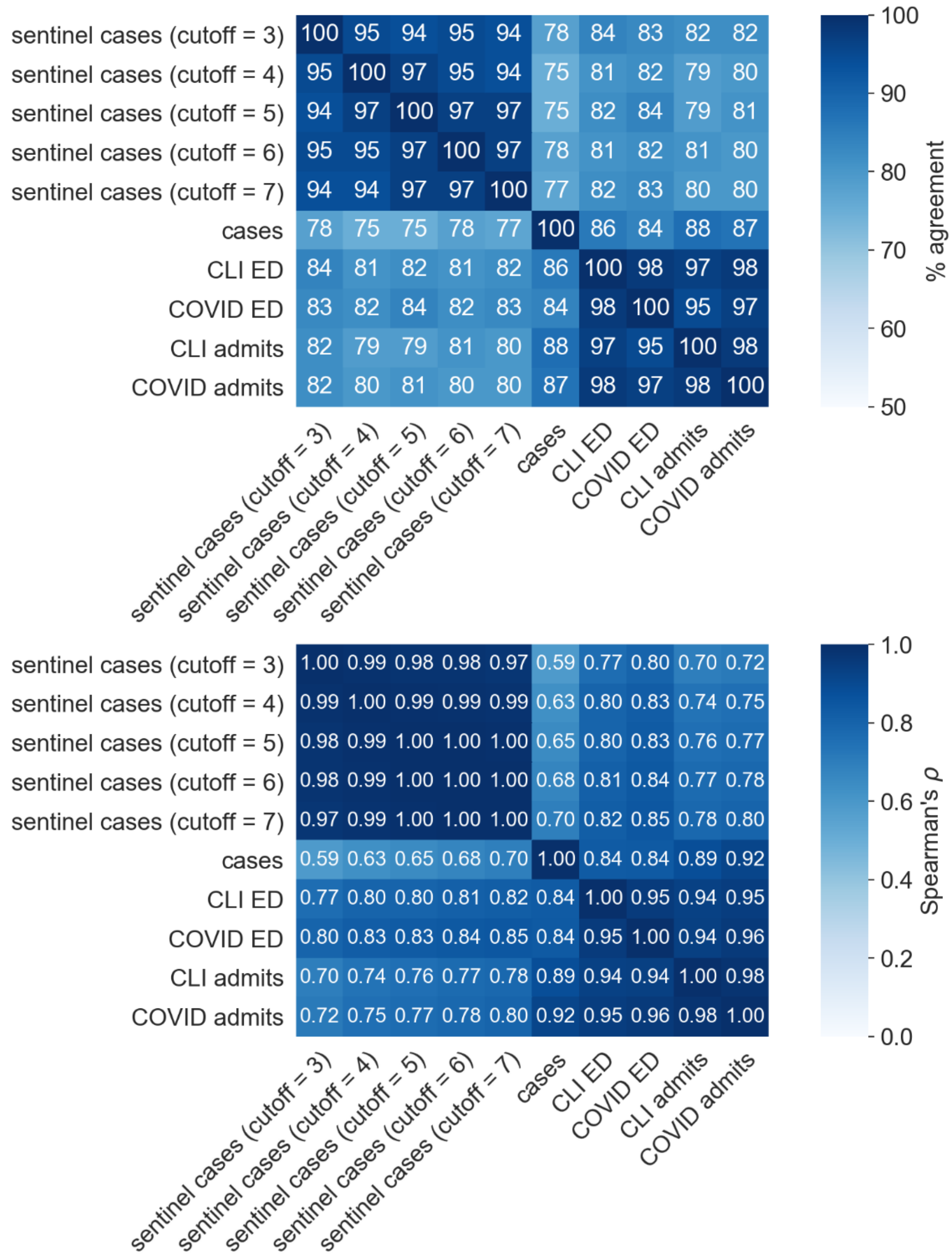

Figure S12: Similarity matrix of percent agreement and Spearman's ρ between $R(t)$ series. Cutoff indicates the highest allowed value for number of days elapsed between symptom onset and specimen collection in order for a specimen to be considered a sentinel sample. A cutoff value of four days was employed elsewhere in the study. Percent agreement is the percentage of dates when the median $R(t)$ estimates of two series are both ≥ 1.0 or both < 1.0.

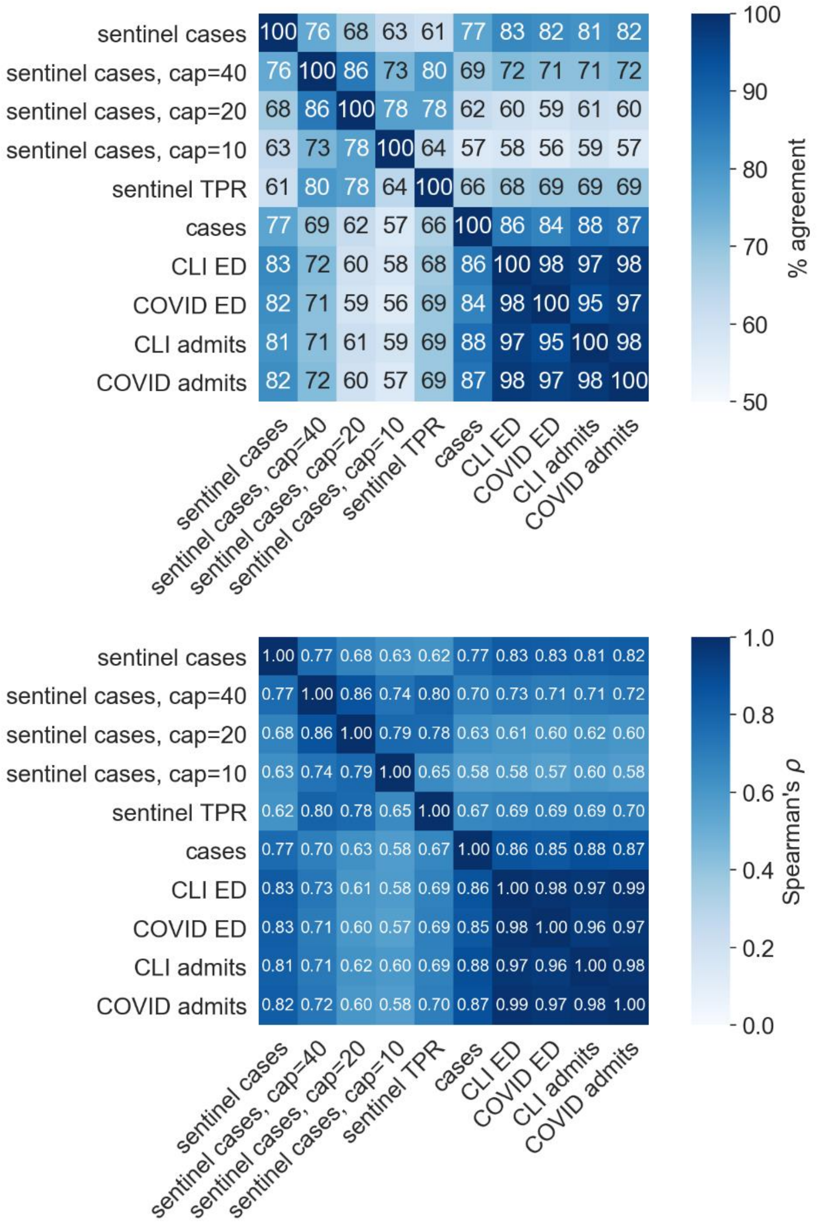

Figure S13: Similarity matrix of percent agreement and Spearman's ρ between $R(t)$ series. "Cap=X" indicates a subsampling technique wherein only sentinel cases from a random sample of X sentinel samples collected each day were considered. Percent agreement is the percentage of dates when the median $R(t)$ estimates of two series are both ≥ 1.0 or both < 1.0.

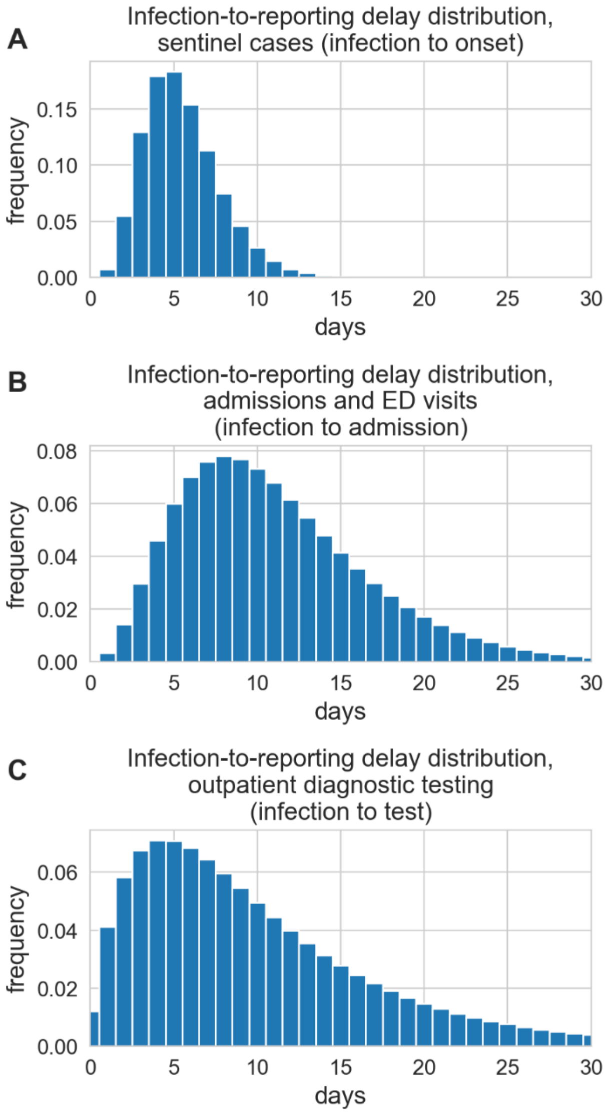

Figure S14: Reporting delay distributions used for $R(t)$ estimation. The time from infection to symptom onset **(A)** was approximated with a gamma distribution with shape factor 5.807 and scale factor 0.948 (mean 5.51 days) [21]. The time from infection to hospitalization or emergency department visit **(B)** was approximated with a gamma distribution with shape factor 3.667 and scale factor 3.029 (mean 11.11 days) [18]. The time from infection to test was approximated with epyestim's default reporting delay distribution (mean 10.33 days) [27, 28]. These distributions are based upon research conducted before the global emergence of the Delta variant, which limits their accuracy as the proportion of cases attributable to Delta increased toward the end of the study period, reaching 24% around June 19 2021, after the end of the study.

Table S1: Retrospective timing of major inflection points in *R*(*t*) curves.

Peak of Fall 2020 wave

**Indicator**	***R*(*t*) crossing date (retrospective)**	**Relative to sentinel cases**
Sentinel cases	Nov 18, 2020	-
Sentinel samples	Nov 20, 2020	2 days
Sentinel TPR	Nov 2, 2020	-16 days
cases	Nov 12, 2020	-6 days
CLI ED visits	Nov 13, 2020	-5 days
COVID-19 ED visits	Nov 14, 2020	-4 days
CLI admissions	Nov 12, 2020	-6 days
COVID-19 admissions	Nov 12, 2020	-6 days

Valley preceding Spring 2021 wave

**Indicator**	***R*(*t*) crossing date (retrospective)**	**Relative to sentinel cases**
Sentinel cases	Feb 22, 2021	-
Sentinel samples	Feb 20, 2021	-2 days
Sentinel TPR	Mar 5, 2021	11 days
cases	Feb 21, 2021	-1 days
CLI ED visits	Feb 28, 2021	6 days
COVID-19 ED visits	Mar 4, 2021	10 days
CLI admissions	Feb 25, 2021	3 days
COVID-19 admissions	Feb 27, 2021	5 days

Peak of Spring 2021 wave

Indicator	***R*(*t*) crossing date (retrospective)**	**Relative to sentinel cases**
Sentinel cases	Apr 19, 2021	-
Sentinel samples	April 22, 2021	3 days
Sentinel TPR	Mar 26, 2021	-24 days
cases	Apr 8, 2021	-11 days
CLI ED visits	Apr 15, 2021	-4 days
COVID-19 ED visits	Apr 15, 2021	-4 days
CLI admissions	Apr 13, 2021	-6 days
COVID-19 admissions	Apr 13, 2021	-6 days
