# Supplementary material for: Tracking changes in SARS-CoV-2 transmission with a novel outpatient sentinel surveillance system in Chicago, USA
Source: Nat Commun. 2022 Sep 22;13:5547. doi: 10.1038/s41467-022-33317-6 (PMC9499975; doi:10.1038/s41467-022-33317-6)
Supplement: Supplementary file 3 — Reporting Summary [file 41467_2022_33317_MOESM3_ESM.pdf]

Corresponding author(s): Jaline Gerardin

Last updated by author(s): Sep 1, 2022

Reporting Summary

Nature Portfolio wishes to improve the reproducibility of the work that we publish. This form provides structure for consistency and transparency in reporting. For further information on Nature Portfolio policies, see our Editorial Policies and the Editorial Policy Checklist.

Statistics

For all statistical analyses, confirm that the following items are present in the figure legend, table legend, main text, or Methods section.

n/a	Confirmed	
☐	☒	The exact sample size (*n*) for each experimental group/condition, given as a discrete number and unit of measurement
☐	☒	A statement on whether measurements were taken from distinct samples or whether the same sample was measured repeatedly
☐	☒	The statistical test(s) used AND whether they are one- or two-sided<br>*Only common tests should be described solely by name; describe more complex techniques in the Methods section.*
☐	☒	A description of all covariates tested
☐	☒	A description of any assumptions or corrections, such as tests of normality and adjustment for multiple comparisons
☐	☒	A full description of the statistical parameters including central tendency (e.g. means) or other basic estimates (e.g. regression coefficient) AND variation (e.g. standard deviation) or associated estimates of uncertainty (e.g. confidence intervals)
☐	☒	For null hypothesis testing, the test statistic (e.g. *F*, *t*, *r*) with confidence intervals, effect sizes, degrees of freedom and *P* value noted<br>*Give P values as exact values whenever suitable.*
☒	☐	For Bayesian analysis, information on the choice of priors and Markov chain Monte Carlo settings
☒	☐	For hierarchical and complex designs, identification of the appropriate level for tests and full reporting of outcomes
☐	☒	Estimates of effect sizes (e.g. Cohen's *d*, Pearson's *r*), indicating how they were calculated

Our web collection on statistics for biologists contains articles on many of the points above.

Software and code

Policy information about availability of computer code

Data collection	No software was used in the collection of data for this study.
Data analysis	All code used for data analysis is available at https://github.com/numalariamodeling/chicago_sentinel_surveillance (doi.org/10.5281/zenodo.7041699)

For manuscripts utilizing custom algorithms or software that are central to the research but not yet described in published literature, software must be made available to editors and reviewers. We strongly encourage code deposition in a community repository (e.g. GitHub). See the Nature Portfolio guidelines for submitting code & software for further information.

Data

Policy information about availability of data

All manuscripts must include a data availability statement. This statement should provide the following information, where applicable:

- Accession codes, unique identifiers, or web links for publicly available datasets
- A description of any restrictions on data availability
- For clinical datasets or third party data, please ensure that the statement adheres to our policy

Restrictions apply to the availability of sentinel surveillance data and individual-level diagnostic tests from I-NEDSS, which contain identifiable private health information. Interested parties should complete CDPH (https://www.chicago.gov/city/en/depts/cdph/provdrs/health_data_and_reports/svcs/data-request-form.html) or IDPH (https://dph.illinois.gov/content/dam/soi/en/web/idph/files/forms/formsoppsdischarge-data-request-form.pdf) data request forms to inquire about access to the I-NEDSS database and data use agreements; IDPH and CDPH will determine access on a case-by-case basis. Public data on cases, testing, ED visits, and hospital admissions are available from CDPH's Public Data Portal (tests: https://data.cityofchicago.org/Health-Human-Services/COVID-19-Cases-Tests-

and-Deaths-by-ZIP-Code/yhhz-zm2v, cases and hospitalizations: https://data.cityofchicago.org/Health-Human-Services/COVID-19-Daily-Cases-Deaths-and-Hospitalizations/naz8-j4nc, ED visits: https://data.cityofchicago.org/Health-Human-Services/COVID-Like-Illness-CLI-and-COVID-19-Diagnosis-Emer/qwib-edaw)

Field-specific reporting

Please select the one below that is the best fit for your research. If you are not sure, read the appropriate sections before making your selection.

☐ Life sciences ☒ Behavioural & social sciences ☐ Ecological, evolutionary & environmental sciences

For a reference copy of the document with all sections, see nature.com/documents/nr-reporting-summary-flat.pdf

Behavioural & social sciences study design

All studies must disclose on these points even when the disclosure is negative.

Study description	The study was a quantitative secondary analysis of public health surveillance data (diagnostic testing data) in the city of Chicago, USA to examine the relationship between this data and traditional indicators of SARS-CoV-2 transmission.
Research sample	The research sample was symptomatic residents presenting for SARS-CoV-2 diagnostic testing at a city- or state-sponsored testing site in the city of Chicago, USA. This sample was chosen for its hypothesized ability to reflect transmission trends in the general population. The sample was not demographically representative of the general population.
Sampling strategy	A convenience sampling procedure was used. Surveillance data had been previously been collected at Chicago Department of Public Health or Illinois Department of Public Health sponsored testing sites in the city of Chicago. In total, 13,952 sentinel samples were collected. As this sample size is on the same order of scale as the number of COVID-19 hospital admissions in Chicago in the study period (n = 14,477), the authors found this sample size appropriate for comparing transmission trends derived from our sample and COVID-19 admissions.
Data collection	All sites, mobile and static, solely offered anterior nares molecular (PCR) diagnostic tests for SARS-CoV-2 infection. All individuals receiving a test were asked to report recent symptoms and provide the date of symptom onset. Those testing at IDPH sites were asked to report the presence or absence of symptoms from COVID-19 symptom list from the Centers for Disease Control and Prevention (CDC). Those testing at CDPH sites were asked only to report the presence or absence of any symptoms without reference to any list of expected symptoms of COVID-19. Specimens were collected at testing sites and transported to an off-site laboratory for processing via PCR. Testing vendors notified patients of results electronically as soon as results were available. During the study period, the median turn-around time from specimen collection to result notification was 2 days, with 95% of tests turned around between 1 and 4 days. Data was pulled on July 6, 2021. As our study retrospectively utilized data from public diagnostic testing sites, researchers were not physically present during specimen collection.
Timing	September 27, 2020 to June 13, 2021
Data exclusions	Individuals reporting for testing who were not Chicago residents, were not symptomatic, did not have a valid date of symptom onset, or their symptom onset date was >= 4 days before specimen collection, were excluded. In total, of 324,872 specimens collected at the public testing sites, 310,920 were excluded from further analysis.
Non-participation	No participants declined participation.
Randomization	No experimental treatment or group allocation was used. Therefore, no randomization protocol was applicable to the design of this study.

Reporting for specific materials, systems and methods

We require information from authors about some types of materials, experimental systems and methods used in many studies. Here, indicate whether each material, system or method listed is relevant to your study. If you are not sure if a list item applies to your research, read the appropriate section before selecting a response.

Materials & experimental systems

n/a	Involved in the study	
☒	☐	Antibodies
☒	☐	Eukaryotic cell lines
☒	☐	Palaeontology and archaeology
☒	☐	Animals and other organisms
☒	☐	Human research participants
☒	☐	Clinical data
☒	☐	Dual use research of concern

Methods

n/a	Involved in the study	
☒	☐	ChIP-seq
☒	☐	Flow cytometry
☒	☐	MRI-based neuroimaging
